# S154. THE ROLE OF DOPAMINE IN PROCESSING THE MEANINGFUL INFORMATION OF OBSERVATIONS, AND IMPLICATIONS FOR THE ABERRANT SALIENCE HYPOTHESIS OF SCHIZOPHRENIA

**DOI:** 10.1093/schbul/sby018.941

**Published:** 2018-04-01

**Authors:** Matthew Nour, Tarik Dahoun, Philipp Schwartenbeck, Rick Adams, Thomas FitzGerald, Christopher Coello, Matthew Wall, Raymond Dolan, Oliver Howes

**Affiliations:** 1 King’s College London, Institute of Psychiatry; 2 Imperial College London, Institute of Clinical Sciences; 3 University College London, Wellcome Trust Centre for Neuroimaging and Max Planck UCL Centre for Computational Psychiatry and Ageing Research; 4 University College London, Institute of Cognitive Neuroscience and Division of Psychiatry; 5 University of East Anglia; 6 University of Oslo, Institute of Basic Medical Sciences; 7 Imanova Centre for Imaging Sciences, Hammersmith Hospital; 8 King’s College London, Institute of Psychiatry, Imperial College London, Institute of Clinical Sciences, MRC London Institute of Medical Sciences

## Abstract

**Background:**

The aberrant salience hypothesis of schizophrenia proposes that symptoms such as paranoia arise when behavioural salience is attributed to neutral stimuli. Mesolimbic dopamine dysfunction is thought to be central to this mechanism; building on findings that activity in this pathway conveys a (signed) reward prediction error signal. Given that many psychotic symptoms are not explicitly related to reward learning, it is relevant that recent studies in rodents have demonstrated a role for midbrain dopamine neurons in value-neutral associative learning. Direct evidence for this role in humans, however, is lacking.

In this study we asked whether the mesolimbic dopamine circuit is involved in encoding the value-neutral meaningful information of observations, using a model-based functional magnetic resonance imaging (fMRI) task and dopamine positron emission tomography (PET). We define ‘meaningful information’ as the degree to which an observation results in a belief-update to an agent’s internal model of the environment (Kullback-Leibler divergence from prior to posterior beliefs; ‘Bayesian surprise’).

**Methods:**

Participants were tasked to infer the current (hidden) state of the environment, using partially-informative observations at each trial, and then report their belief at the end of each trial. Participant beliefs were modelled using a Hidden Markov Model of the task and iterative application of Bayes’ rule, allowing us to quantify the Bayesian surprise (meaningful information content) associated with a trial observation. Crucially, our task de-correlated Bayesian surprise from both the pure sensory unexpectedness of an observation (unexpected but meaningless information) and its signed reward prediction error. 39 healthy participants (22M, mean age 26y) performed 180 task trials within an fMRI scanner. 36 participants also had a [11C]-(+)-4-propyl-9-hydroxy-naphthoxazine (PHNO) PET scan to quantify dopamine-2/3 receptor (D2/3R) availability. 17 participants additionally had a second PET scan 3hrs post 0.5mg/kg oral dexamphetamine, to quantify striatal dopamine release capacity. Neuroimaging analyses were restricted to the bilateral substantia nigra/ventral tegmental area (SN/VTA) and ventral striatum (VS).

**Results:**

Our computational model closely predicted participant behaviour (R2= .67), and there was a negative correlation between subclinical paranoia and the degree to which participant behaviour approximated normative Bayesian performance (rho = -.60, P<0.001). Neuronal activation encoding the meaningful information content of an observation (Bayesian surprise) was present in SN/VTA and VS (both P(peak)<0.05, SVC), whereas no such encoding was present for sensory unexpectedness or reward-prediction error. Crucially, activation encoding Bayesian surprise was inversely correlated with D2/3R availability in the SN/VTA (rho = -.43, P=0.009), consistent with a tonic inhibitory role for midbrain D2/3Rs. Moreover, activation encoding Bayesian surprise was inversely related to dopamine release capacity in the VS (rho = -.66, P=0.005), indicating that subjects with high dopamine release capacity showed blunted striatal activation in response to belief-changing information, as is also found in schizophrenia.

**Discussion:**

We provide direct evidence in humans that a mesolimbic dopamine circuit is involved in encoding the meaningful information content of observations, distinct from its involvement in processing signed reward prediction error. These results implicate dopamine in a wider range of function than reward learning, including updating a predictive associative model of the world, and are therefore relevant for the aberrant salience hypothesis of schizophrenia.

